# Preoperative Neutrophil to Lymphocyte Ratio, Platelet to Lymphocyte Ratio, and Mean Platelet Volume as Predictors of 1-Year Mortality in Patients Undergoing an Open Repair of Abdominal Aortic Aneurysms: A Retrospective Study

**DOI:** 10.3390/jcm10225410

**Published:** 2021-11-19

**Authors:** Da Eun Ko, Hei Jin Yoon, Sang Beom Nam, Suk Won Song, Gisong Lee, Sung Yeon Ham

**Affiliations:** 1 Department of Anesthesiology and Pain Medicine, Yonsei University College of Medicine, Seoul 06273, Korea; DANA94K@yuhs.ac (D.E.K.); PHIN86@yuhs.ac (H.J.Y.); SBNAM@yuhs.ac (S.B.N.); GISONGLEE@yuhs.ac (G.L.); 2 Anesthesia and Pain Research Institute, Yonsei University College of Medicine, Seoul 06273, Korea; 3 Department of Thoracic and Cardiovascular Surgery, Yonsei University College of Medicine, Seoul 06273, Korea; sevraphd@yuhs.ac

**Keywords:** neutrophil to lymphocyte ratio, platelet to lymphocyte ratio, mean platelet volume, aortic surgery, abdominal aortic aneurysm

## Abstract

Objectives: To investigate if preoperative neutrophil to lymphocyte ratio (NLR), platelet to lymphocyte ratio (PLR), or mean platelet volume (MPV) could be used to predict 1-year mortality in patients undergoing open abdominal aortic aneurysm (AAA) repair. Methods: We retrospectively reviewed 382 patients who underwent open AAA repair between January 2008 and July 2019. We divided the patients into two groups based on 1-year mortality and compared the preoperative NLR, PLR, and MPV. The patients were then classified into tertiles based on their preoperative NLR (first tertile: <2.41 (*n* = 111); second tertile: 2.41 ≤ NLR ≤ 6.07 (*n* = 111); and third tertile: >6.07 (*n* = 112)). We compared the incidence of mortality and morbidity across the aforementioned tertiles. We performed a stepwise logistic regression analysis to evaluate the predictors for mortality. An additional subgroup analysis was performed by dividing the cases into non-ruptured and ruptured cases. Results: The preoperative NLR was significantly higher in the non-survivor group than in the survivor group (10.53 ± 7.60 vs. 5.76 ± 6.44, respectively, *p* = 0.003). The PLR and MPV were similar between the groups (145.35 ± 91.11 vs. 154.20 ± 113.19, *p* = 0.626, 9.38 ± 1.20 vs. 9.11 ± 1.39, *p* = 0.267, respectively). The incidence of 1-year mortality was 2.7%, 9.0%, and 14.3% in the first, second, and third NLR tertiles, respectively (*p* = 0.009). Higher NLR (odds ratio 1.085, 95% confidence interval 1.016–1.159, *p* = 0.015) and ruptured AAA (odds ratio 2.706, 95% confidence interval 1.097–6.673, *p* = 0.031) were the independent predictors of 1-year mortality in all patients. Moreover, the preoperative NLR was significantly higher in the ruptured AAA than in the non-ruptured AAA group (11.17 ± 7.90 vs. 4.10 ± 4.75, *p* < 0.001). In subgroup analysis, preoperative NLR (odds ratio 1.144, 95% confidence interval 1.031–1.271, *p* = 0.012) and PLR (odds ratio 0.986, 95% confidence interval 16 0.975–0.998, *p* = 0.017) was an independent predictor for 1-year mortality in ruptured cases. Conclusions: We demonstrated an independent relationship between the preoperative NLR and 1-year mortality in patients undergoing open AAA repair, besides PLR and MPV. Furthermore, the NLR and PLR had predictive power for 1-year mortality in ruptured cases.

## 1. Introduction

Abdominal aortic aneurysm (AAA) is a multifactorial degenerative disorder, which if untreated can lead to catastrophic complications [1]. The treatment for AAA includes open and endovascular repair, both of which carry a significant degree of risk [2,3]. Furthermore, AAA accompanies several serious comorbidities such as advanced age, hypertension, diabetes mellitus (DM), hyperlipidemia and smoking, which can worsen prognosis [3,4]. Thus, researchers have performed several studies addressing simple and readily available risk stratification markers, such as complete blood count in patients undergoing open AAA repair [5,6,7].

The neutrophil to lymphocyte ratio (NLR) has been frequently used as a marker of the systemic inflammatory response, which reflects neutrophilia and lymphopenia [8]. The primary pathophysiology of AAA involves chronic inflammation in the aortic wall and atherosclerosis, accompanied by thrombosis [9]. NLR was proposed as a fair indicator of poor prognosis in patients with AAA [6,10,11]. The mean platelet volume (MPV) is a marker of platelet activation and an indicator of the activation of thrombus formation. Moreover, it is reportedly associated with the prognosis of patients with cardiovascular diseases [12]. Moreover, the platelet to lymphocyte ratio (PLR) suggests thrombosis and inflammation and indicates a high risk of cardiovascular events in various groups of patients [13]. The PLR is associated with poor prognosis following AAA repair [5]. NLR, PLR and MPV have also been investigated in other, non-vascular diseases such as obesity, DM, and nonalcoholic fatty liver disease (NAFLD). Platelet indices have also been reported to have a predictive role for fibrosis [14]. Despite accumulating evidence for the prognostic value of white blood cell counts in abdominal aortic aneurysms [6,10,15], few studies have investigated the value of these parameters, including NLR, MPV and PLR in patients undergoing AAA open repair.

We aimed to assess the NLR, MPV and PLR as valuable predictors of perioperative mortality in patients undergoing open repair for AAA.

## 2. Materials & Methods

### 2.1. Study Population

**Figure 1:**
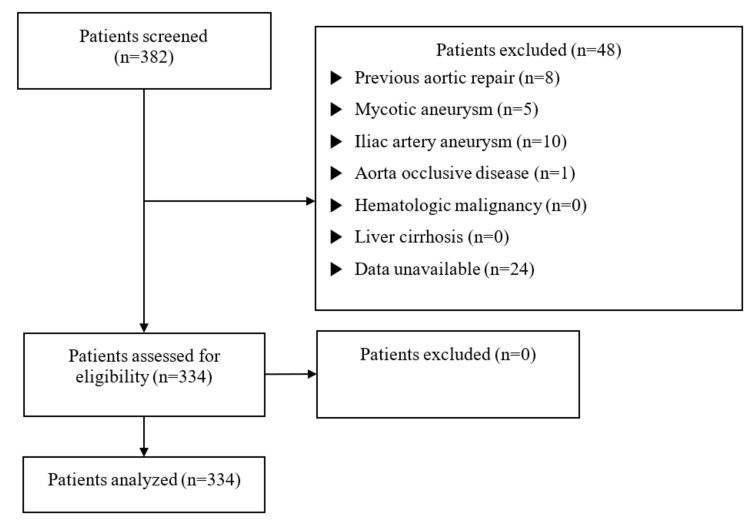
Flowchart depicting patient enrollment.

Following approval by the institutional review board of the Yonsei University Health System, Seoul, South Korea (IRB protocol No. 3-2020-0479), the requirement for informed consent was waived considering the retrospective nature of this study. The study comprised 382 patients who underwent an AAA open repair at the Gangnam Severance hospital between January 2008 and July 2019. Patients with previous aortic repair within 6 months (*n* = 8), mycotic aneurysm (*n* = 5), iliac artery aneurysm (*n* = 10), aorta occlusive disease (*n* = 1), hematologic malignancy (*n* = 0), liver cirrhosis (*n* = 0), or incomplete laboratory data (*n* = 24) were excluded from the analysis. We eventually analyzed 334 patients (Figure 1). We divided the patients into survivors and non-survivors, and analyzed three classified groups based on their preoperative NLR levels (first tertile: NLR < 2.41 (*n* = 111); second tertile: 2.41 ≤ NLR ≤6.07 (*n* = 111); and third tertile: NLR > 6.07 (*n* = 112)).

### 2.2. Demographic and Clinical Data

Patient demographic and clinical data included their sex, age, body mass index (BMI), comorbidities (hypertension, diabetes mellitus, cerebrovascular accident (CVA), coronary artery occlusive disease, chronic obstructive pulmonary disease, and chronic kidney disease), and smoking. Preoperative steroid use was recorded. Perioperative laboratory data comprised the complete blood count, NLR, MPV, PLR, prothrombin time (PT), C-reactive protein (CRP), B-type natriuretic peptide, creatinine, blood urea nitrogen (BUN), and estimated glomerular filtration rate (eGFR). We recorded the etiology of the disease, intake and output during surgery, and the total duration of surgery. The incidence of postoperative complications, including intensive care unit (ICU) readmission, reoperation, the requirement of renal replacement therapy, pulmonary complications, infection, acute kidney injury, myocardial infarction, cerebrovascular accidents, and mortality was collected. Moreover, we recorded the duration of hospitalization and ICU stay.

### 2.3. Study Endpoints

The incidence of 1-year mortality after AAA open repair was the primary endpoint. By contrast, the secondary outcome comprised the incidence of postoperative complications including infections, pulmonary complication, myocardial infarction, CVA, and acute kidney injury within 48 h following the surgery. Myocardial infarction was defined as an increase in the peak serum troponin-T isozyme >5 times the upper limit of normal. CVA was defined as the presence of recent neurological deterioration induced by embolic, thrombotic, or hemorrhagic brain injury. Infection was defined as the occurrence of one of the following conditions: pneumonia, sepsis, peritonitis, urinary tract infection, or graft infection. Pulmonary complication was defined as respiratory failure, an exacerbation of chronic obstructive pulmonary disease, or culture-negative pneumonia that required prolonged mechanical ventilation (>24 h). Acute kidney injury within 48 h following surgery was defined using the Kidney Disease Improving Global Guidelines Clinical Practice Guidelines.

### 2.4. Statistical Analyses

SPSS version 23 (IBM Corp, Armonk, NY, USA) was used to perform all statistical analyses. All results are described as means ± standard deviations or the number of patients (%). We assessed the normality using the Kolmogorov-Smirnov test. We conducted independent *t*-tests or Mann-Whitney U tests to compare continuous variables. While one-way analysis of variance or Kruskal-Wallis tests were used to compare the three groups, Bonferroni’s method was used for post-analysis. We compared the categorical variables using the chi-square test or Fisher’s exact test. Moreover, we performed a logistic regression analysis to evaluate the predictors for 1-year mortality. For the multivariate analysis, we performed a stepwise selection method, and selected variables with *p* < 0.2 in the univariate analysis. Predictability was expressed as the odds ratio (OR) and 95% confidence interval (CI). A *p*-value < 0.05 was considered statistically significant.

## 3. Results

### 3.1. Baseline and Perioperative Characteristics

A total of 382 patients were reviewed during the study period, and 334 patients were eventually analyzed (Figure 1). Table 1 summarizes the baseline characteristics of patients, compared to their 1-year mortality. Baseline characteristics, including their sex, age, BMI, and medical history, were similar between the groups. Ruptured AAA was more frequent in the non-survivor group (26.6% vs. 58.6%, respectively, *p* < 0.001) as well as in emergency cases (54.8% vs. 89.7%, respectively, *p* < 0.001). The preoperative white blood cell count and neutrophil count were higher in the non-survivor group than in the survivor group (9661.15 ± 4531.01/μL vs. 14328.62 ± 6821.57/μL, respectively, *p* = 0.001 and 7089.71 ± 4543.53/μL vs. 11833.71 ± 6670.75/μL, respectively, *p* < 0.001). In contrast, the preoperative lymphocyte count was lower in the non-survivor group than in the survivor group (1712.39 ± 814.84/μL vs. 1483.16 ± 689.47/μL, respectively, *p* < 0.001). The non-survivor group revealed lower preoperative hemoglobin levels than the survivor group (12.07 ± 2.61 g/dL vs. 10.39 ± 2.66 g/dL, respectively, *p* = 0.001). Moreover, the preoperative hematocrit was lower in the non-survivor group than in the survivor group (36.25 ± 7.54% vs. 31.39 ± 7.81%, respectively, *p* = 0.001). However, the platelet count was not significantly different between the groups (204,328.31 ± 72,993.61/μL vs. 187,206.90 ± 107,334.38/μL, *p* = 0.407). The preoperative NLR was significantly higher in the non-survivor group than in the survivor group (5.76 ± 6.44 vs. 10.53 ± 7.60, respectively, *p* = 0.003). The MPV and PLR values demonstrated inconsequential differences between the two groups (9.38 ± 1.20 fL vs. 9.11 ± 1.39 fL, respectively, *p* = 0.267, 145.35 ± 91.11 vs. 154.20 ± 113.19, respectively, *p* = 0.626). The non-survivor group had more prolonged PT (INR) and higher preoperative BUN levels than the survivor group (1.11 ± 0.24 vs. 1.47 ± 0.54, respectively, *p* = 0.002 and 20.06 ± 9.69 mg/dL vs. 24.32 ± 10.80 mg/dL, respectively, *p* = 0.028), along with lower eGFR values (59.00 ± 24.87 mL/min/1.73m^2^ vs. 72.89 ± 22.87 mL/min/1.73m^2^, respectively, *p* = 0.024). However, creatinine levels did significantly differ between the groups (1.17 ± 0.82 mg/dL vs. 1.32 ± 0.49 mg/dL, respectively, *p* = 0.323).

Table 2 outlines the baseline characteristics and operation-related data based on preoperative NLR levels across the first, second, and third tertiles. Baseline characteristics were similar among the three groups, except for age (69.08 ± 8.82 vs. 73.07 ± 9.55 vs. 73.30 ± 9.12, respectively, *p* = 0.001) and history of hypertension (76.4% vs. 61.3% vs. 70.5%, respectively, *p* = 0.049). The proportion of ruptured AAA (3.6% vs. 21.6% vs. 62.5%, respectively, *p* < 0.001) and emergency surgery (25.2% vs. 52.3% vs. 95.5%, respectively, *p* < 0.001) was significantly different among the tertiles. The third tertile group received more packed red blood cells and fresh frozen plasma transfusions than any other group (1.07 ± 2.01 vs. 1.50 ± 2.54 vs. 3.34 ± 4.26 packs, *p* < 0.001, 0.77 ± 1.71 vs. 1.02 ± 2.06 vs. 2.27 ± 3.10 packs, *p* < 0.001, respectively).

Table 3 summarizes postoperative morbidity and mortality across the NLR tertiles. While the duration of hospital stay was significantly different across the tertiles (14.02 ± 13.97 vs. 20.29 ± 21.72 vs. 19.65 ± 15.31, respectively, *p* = 0.005), the duration of ICU stay was similar among the groups (6.04 ± 61.20 vs. 3.49 ± 5.64 vs. 6.43 ± 9.25, respectively, *p* = 0.805) The reintubation rate was the highest in the third tertile group. (6.4% vs. 10.3% vs. 20.0%, respectively, *p* = 0.007) The proportion of patients with mechanical ventilation >24 h was the highest in the third tertile group. (6.3% vs. 10.8% vs. 30.4%, respectively, *p* < 0.001). Re-operation because of postoperative bleeding was most frequent in the third tertile group (0.9% vs. 1.8% vs. 7.1%, respectively, *p* = 0.046). Patients in the highest NLR tertile reported more pulmonary complications and postoperative infections (7.2% vs. 19.8% vs. 24.1%, *p* = 0.002, 3.6% vs. 7.2% vs. 15.2%, *p* = 0.007, respectively). Moreover, the third tertile group comprised the highest in-hospital mortality of any group (2.7% vs. 9.0% vs. 13.4%, respectively, *p* = 0.015). We observed similar results for 1-month mortality and 1-year mortality (1.8% vs. 6.3% vs. 10.7%, *p* = 0.023, 2.7% vs. 9.0% vs. 14.3% *p* = 0.009, respectively).

### 3.2. Independent Prognostic Significance of NLR, MPV, and PLR in Predicting 1-Year Mortality after AAA Open Repair

Table 4 summarizes the logistic regression analysis for the predictors of 1-year mortality after AAA open repair. In the univariate analysis, ruptured AAA, preoperative NLR, PLR, and MPV revealed a difference of *p* < 0.2 and were selected for the multivariate analysis. Ruptured AAA (OR 2.706, 95% CI 1.097–6.673, *p* = 0.031) and preoperative NLR (OR 1.085, 95% CI 1.016–1.159, *p* = 0.015) remained the independent predictors for 1-year mortality after AAA open repair. To verify the prognostic impact of NLR, an additional subgroup analysis was performed by dividing the cases into non-ruptured and ruptured cases. Preoperative NLR (OR 1.144, 95% CI 1.031–1.271, *p* = 0.012) and PLR (OR 0.986, 95% CI 0.975–0.998, *p* = 0.017) were independent predictors for 1-year mortality in ruptured cases.

### 3.3. Baseline and Perioperative Characteristics Depending on the Presence of Rupture

Table 5 outlines the baseline laboratory data stratified according to the presence of rupture in patients with AAA. In ruptured AAA cases, the preoperative white blood cell count and neutrophil count were higher than those in non-ruptured cases. (14,442.14 ± 5960.32/μL vs. 8249.36 ± 2931.44/μL, *p* < 0.001, 12,154.97 ± 5628.89/μL vs. 5569.29 ± 2947.05/μL, *p* < 0.001, respectively). By contrast, the lymphocyte count was significantly lower in the ruptured cases (1374.01 ± 670.37/μL vs. 1824.74 ± 822.26/μL, respectively, *p* < 0.001). While the preoperative NLR and MPV was higher in ruptured AAA than in non-ruptured AAA cases (11.17 ± 7.90 vs. 4.10 ± 4.75, *p* < 0.001, 9.59 ± 1.24 vs. 9.26 ± 1.19, *p* = 0.023, respectively), the PLR did not significantly differ between the groups (142.22 ± 84.14 vs. 155.52 ± 111.62, respectively, *p* = 0.291). The hemoglobin, hematocrit, and platelet levels were significantly lower in patients with ruptured AAA (12.85 ± 2.13 g/dL vs. 9.70 ± 2.47 g/dL, *p* < 0.001, 38.47 ± 6.13% vs. 29.47 ± 7.30%, *p* < 0.001, 217,017.52 ± 71,052.99/μL vs. 168,704.08 ± 78,781.86/μL, *p* < 0.001, respectively). Moreover, they demonstrated prolonged PT (INR) and higher preoperative lactate levels than those with non-ruptured AAA (1.06 ± 0.16 mmol/L vs. 1.35 ± 0.42 mmol/L, *p* < 0.001 and 0.97 ± 0.41 vs. 4.45 ± 2.66, *p* < 0.001, respectively).

## 4. Discussion

We investigated the relationship between the NLR, MPV, and PLR and 1-year mortality in patients who underwent an AAA open repair. High preoperative NLR was an independent risk factor for predicting 1-year mortality in patients with AAA along with ruptured cases. Both MPV and PLR were not significantly associated with mortality. According to a subgroup analysis based on the presence of ruptures, preoperative NLR and PLR were independent risk factors in ruptured cases, and no significant risk factors were found in non-rupture cases.

Neutrophilia and lymphopenia are physiologic responses of the innate immune system to various stressful insults, including systemic inflammation, injury, and stress [8]. Neutrophilia is reportedly observed during myocardial injury as a part of an acute inflammatory reaction [16]. Along with inflammation, neutrophilia is associated with platelet activation and atherothrombosis [17,18]. Lymphopenia is induced by hormones, cytokines, and chemokines and reflects the strength and intensity of a stressful event [8]. Moreover, it represents immune-depressed conditions in cases of increased lymphocyte apoptosis and has been utilized as an indicator of poor prognosis in patients with coronary heart disease [19]. Considering that both neutrophilia and lymphopenia increase the NLR, elevated NLR is correlated to poor prognosis. The previous literature has described a strong relationship between high NLR and poor outcomes in patients with acute coronary syndrome [16], coronary bypass graft [20], and acute aortic dissection [21]. Appleton et al. reported that preoperative NLR > 5 increased both 30-day mortality and long-term outcomes in open AAA surgery [6]. Moreover, NLR > 5 has been identified as an independent marker of 30-day morbidity in ruptured AAA [10]. In accordance with previous results, the preoperative NLR value was higher in the mortality group than in the survivor group. Moreover, the NLR had a prognostic impact on 1-year mortality in patients undergoing AAA open repair.

MPV is a biomarker of platelet activity and has been used as prognostic marker for adverse outcomes and mortality in various cohorts, such as coronary artery disease [12] and type A acute aortic dissection [22]. Considering that platelet activation plays a key role in atherothrombosis [23], MPV may exert a prognostic impact in patients with aortic disease. Furthermore, MPV has been associated with aortic distensibility and chronic inflammation in patients with stable coronary artery disease [24]. However, the preoperative MPV was not significantly different between the survivors and non-survivors and failed to act as an independent predictor of 1-year mortality in patients undergoing AAA open repair. By contrast, we demonstrated that the MPV was significantly higher in ruptured cases than in non-ruptured cases. A possible explanation for this discrepancy is that platelets play an important role in ruptured cases. However, they are relatively unrelated to chronic inflammation, the primary pathogenesis of AAA. However, the possibility of a relatively small size being required to reach statistical significance or the existence of confounding factors necessitates further investigation.

The PLR is obtained by dividing the platelet count by the lymphocyte count, hence reflecting both thrombosis and inflammation [25]. Since PLR reflects inflammation, atherosclerosis, and platelet activation, previous researchers have reported on the prognostic impact of PLR in patients with various cardiovascular diseases [13,25]. The PLR levels at admission were associated with in-hospital mortality in patients with type A acute aortic dissection [21]. It is difficult to compare our findings with previous results since there are limited reports on the role of PLR in patients with AAA. A previous study demonstrated that extreme PLR values are associated with the risk of complications in patients undergoing AAA surgical repair [5]. Although PLR failed to predict 1-year mortality in all patients, PLR displayed a predictive power in patients with ruptured AAA in the current study. A possible explanation for the discrepancy was the occurrence of platelet consumption in the ruptured cases.

AAA is a progressive pathological dilatation of the aortic wall, characterized by chronic inflammation and atherothrombosis [9,26]. According to Kuivaniemie et al., theories of AAA pathogenesis include inflammation, smooth muscle cell apoptosis, extracellular matrix degradation and oxidative stress [27]. Furthermore, chronic inflammation also plays a central role in AAA progression [28,29]. Previous studies have reported that inflammatory cells, including T cells, macrophages, neutrophils, and mast cells infiltrate into the aortic walls, thus initiating and promoting AAA expansion [30]. Likewise, Dawson et al. reported that aortic aneurysm secret proinflammatory cytokine interleukin-6 and elevated circulating IL-6 were independent risk factors for cardiovascular mortality [31,32]. Therefore, higher NLR can supposedly predict poor prognosis in patients with AAAs. We suggested that MPV does not have prognostic value compared to NLR, considering that inflammation plays a key role in AAA development and progression.

However, CRP, which reflects systemic inflammation, was not significantly associated with mortality in patients with AAA. Previous studies on plasma cytokine levels in AAA reported a positive linear correlation between CRP levels and the aortic diameter [33,34]. Thus, elevated CRP in AAA disproves the involvement of inflammation in the development of AAA [35]. However, the association between CRP and AAA progression is unclear [36,37]. We observed no significant difference in CRP levels between the survivors and non-survivors, and between non-ruptured and ruptured AAA groups, in contrast to NLR results. CRP was primarily related to the development rather the expansion of AAA [37]. Thus, it may not have prognostic value as a predictor in patients with AAA. Additionally, this difference may be attributed not only to inflammation but also to platelet activation, atherothrombosis [17,18], and the intensity of stressful events, [8] considering that NLR involves both neutrophilia and lymphopenia.

The fact that NLR and PLR could predict prognosis in ruptured cases but not in non-ruptured cases means that inflammation and platelet consumption are more likely to occur in ruptured cases with mortality. This can be demonstrated by the significantly higher neutrophil and significantly lower lymphocyte and platelet counts in ruptured cases. In a recent study, shock, hemorrhage, and hypertensive disease were related as the cause of death in ruptured aneurysms, but only hypertensive disease was found to be related to unruptured aneurysms [4]. These results are consistent with our study in that NLR and PLR were predictive of 1-year mortality. Moreover, although it failed to obtain statistical significance, the logistic regression analysis showed that hypertension had a tendency to predict death in non-ruptured cases, similar to the results of the previous study. Since the only validated measure of rupture risk is the diameter of an abdominal aortic aneurysm [3], an increased inflammatory marker in ruptured cases may be due to the involvement of inflammation in the expansion of an aneurysm rather than development of an aneurysm. Furthermore, these changes in NLR and PLR in ruptured cases with mortality may be partly due to the inflammation and platelet consumption caused by rupture.

The strengths of our study were that we predominantly investigated the prognostic value of NLR, MPV, and PLR in patients undergoing AAA open repair. Second, we included a relatively longer follow-up period, which has rarely been investigated before.

However, our study had some limitations. Considering the inherent nature of retrospective studies, we could collect existing data alone. Patients without available laboratory data were excluded, thus creating possibilities for selection bias. Second, although we investigated patients’ history of taking steroids and compared them between groups, it could be a limitation that we did not exclude patients taking steroids that could affect complete blood counts. Lastly, we selected all patients undergoing AAA open repair, including ruptured cases. However, patients with elective cases also have a substantial risk of rupture, and since NLR is related to the pathogenesis and progression of AAA rather than simply rupture, we think it is meaningful to analyze patients with abdominal aortic aneurysm across all spectrums including asymptomatic, symptomatic non-ruptured, and symptomatic ruptured cases. Furthermore, we additionally performed a subgroup analysis. Statistical significance may not have been reached in non-ruptured cases due to the small sample size, or due to the relatively low mortality. Furthermore, NLR and ruptured cases were identified as independent risk factors for mortality. This necessitates exploring the prognostic impact of NLR in both non-ruptured and ruptured cases.

## 5. Conclusions

In conclusion, NLR was identified as an independent predictor for 1-year mortality in patients undergoing AAA open repair. In a subgroup analysis, NLR and PLR were predictors of 1-year mortality in ruptured cases, but not in non-ruptured cases. However, MPV did not reveal a prognostic impact. NLR reflects chronic inflammation, which is the primary pathophysiology of developing AAA. Therefore, NLR rather than MPV could have a prognostic impact on patients with AAA.

## Figures and Tables

**Table 1:** Baseline characteristics and laboratory data, stratified according to 1-year mortality.

	Survivor (*n* = 305)	Non-Survivor (*n* = 29)	*p*-Value
Female sex	61 (20.0%)	2 (6.9%)	0.085
Age (years)	71.69 ± 9.42	73.28 ± 8.54	0.382
Height (cm)	165.35 ± 8.25	166.04 ± 8.11	0.389
Weight (kg)	64.51 ± 11.19	64.56 ± 13.30	0.985
BMI	23.51 ± 3.13	23.42 ± 4.52	0.925
Smoking	113 (37.0%)	11 (37.9%)	0.925
HTN	214 (70.4%)	17 (58.6%)	0.189
DM	54 (17.7%)	5 (17.2%)	0.950
CVA	43 (14.1%)	5 (17.2%)	0.586
CAOD	72 (23.7%)	8 (27.6%)	0.638
COPD	7 (2.3%)	1 (3.4%)	0.520
CRF	26 (8.5%)	1 (3.4%)	0.491
ESRD	3 (1.0%)	0	1.000
Rupture	81 (26.6%)	17 (58.6%)	<0.001 *
Emergency	167 (54.8%)	26 (89.7%)	<0.001 *
Preop steriod	5 (1.6%)	0	1.000
Preop WBC (/μL)	9661.15 ± 4531.01	14,328.62 ± 6821.57	0.001 *
Preop neutrophil (/μL)	7089.71 ± 4543.53	11,833.71 ± 6670.75	<0.001 *
Preop lymphocyte (/μL)	1712.39 ± 814.84	1483.16 ± 689.47	<0.001 *
Preop Hb (g/dL)	12.07 ± 2.61	10.39 ± 2.66	0.001 *
Preop Hct (%)	36.25 ± 7.54	31.39 ± 7.81	0.001 *
Preop PLT (/μL)	204,328.31 ± 72993.61	187,206.90 ± 107334.38	0.407
Preop NLR	5.76 ± 6.44	10.53 ± 7.60	0.003 *
Preop MPV (fL)	9.38 ± 1.20	9.11 ± 1.39	0.267
Preop PLR	145.35 ± 91.11	154.20 ± 113.19	0.626
Preop PT (INR)	1.11 ± 0.24	1.47 ± 0.54	0.002 *
Preop procalcitonin (ng/mL)	0.22 ± 0.73	0.45 ± 0.41	0.402
Preop CRP (mg/L)	21.87 ± 43.82	35.84 ± 47.59	0.234
Preop BNP (pg/mL)	166.52 ± 300.09	234.42 ± 264.01	0.488
Preop BUN (mg/dL)	20.06 ± 9.69	24.32 ± 10.80	0.028 *
Preop Cr (mg/dL)	1.17 ± 0.82	1.32 ± 0.49	0.323
Preop eGFR (mL/min/1.73 m^2^)	72.89 ± 22.87	59.00 ± 24.87	0.024 *

Values are presented as means ± standard deviations or number of patients (%). BMI: body mass index, HTN: hypertension, DM: diabetes mellitus, CVA: cerebrovascular accident, CAOD: coronary artery occlusive disease, COPD: chronic obstructive pulmonary disease, CRF: chronic renal failure, ESRD: end stage renal disease, Hb: hemoglobin, Hct: hematocrit, PLT: platelet, NLR: neutrophil to lymphocyte ratio, MPV: mean platelet volume, PLR: platelet to lymphocyte ratio, PT: prothrombin time, CRP: C-reactive protein, BNP: B type natriuretic peptide, BUN: blood urea nitrogen, Cr: creatinine, eGFR: estimated glomerular filtration rate * *p* < 0.05.

**Table 2:** Baseline characteristics and operation related data across three NLR tertiles.

	First Tertile (*n* = 111)	Second Tertile (*n* = 111)	Third Tertile (*n* = 112)	*p*-Value
Female sex	23 (20.7%)	19 (17.1%)	21 (18.8%)	0.790
Age (years)	69.08 ± 8.82	73.07 ± 9.55	73.30 ± 9.12	0.001 *
Height (cm)	164.85 ± 9.22	164.99 ± 7.78	166.38 ± 7.56	0.321
Weight (kg)	65.85 ± 12.04	63.72 ± 11.42	63.94 ± 10.48	0.309
BMI	24.12 ± 3.32	23.37 ± 3.28	22.99 ± 3.05	0.032
Smoking	42 (37.8%)	41 (36.9%)	41 (36.6%)	0.981
HTN	84 (76.4%)	68 (61.3%)	79 (70.5%)	0.049 *
DM	23 (20.7%)	16 (14.4%)	20 (17.9%)	0.467
CVA	18 (16.2%)	13 (11.7%)	17 (15.2%)	0.605
CAOD	27 (24.3%)	32 (29.1%)	21 (18.8%)	0.196
COPD	1 (0.9%)	5 (4.5%)	2 (1.8%)	0.236
CRF	8 (7.2%)	8 (7.2%)	11 (9.8%)	0.710
ESRD	0	1 (0.9%)	2 (1.8%)	0.776
Rupture	4 (3.6%)	24 (21.6%)	70 (62.5%)	<0.001 *
Emergency	28 (25.2%)	58 (52.3%)	107 (95.5%)	<0.001 *
Preop steroid	1 (0.9%)	3 (2.7%)	1 (0.9%)	0.542
Amount of crystalloid (mL)	2875.94 ± 1796.88	2675.95 ± 1675.66	3182.89 ± 1592.65	0.089
Amount of colloid (mL)	442.59 ± 591.56	503.18 ± 615.34	554.09 ± 772.70	0.465
Urine output (mL)	443.70 ± 364.54	427.06 ± 362.91	458.11 ± 458.75	0.851
Cell saver (mL)	443.70 ± 364.54	427.06 ± 362.91	458.11 ± 458.75	0.505
Bleeding (mL)	974.84 ± 881.08	1121.82 ± 1345.87	1356.09 ± 1679.08	0.156
pRBC transfusion (pack)	1.07 ± 2.01	1.50 ± 2.54	3.34 ± 4.26	<0.001 *
FFP (pack)	0.77 ± 1.71	1.02 ± 2.06	2.27 ± 3.10	<0.001 *
PLTconc (pack)	0.76 ± 2.68	0.77 ± 2.87	1.96 ± 4.99	0.062
Operative time (min)	140.22 ± 74.89	135.56 ± 56.73	148.06 ± 65.23	0.361
Anesthesia time (min)	218.77 ± 84.55	203.53 ± 62.35	198.84 ± 71.67	0.108
ACC time (min)	42.03 ± 19.80	36.27 ± 17.21	42.59 ± 22.08	0.066

Values are presented as mean ± standard deviation or number of patients (%). BMI: body mass index, HTN: hypertension, DM: diabetes mellitus, CVA: cerebrovascular accident, CAOD: coronary artery occlusive disease, COPD: chronic obstructive pulmonary disease, CRF: chronic renal failure, ESRD: end stage renal disease, pRBC: packed RBC, FFP: fresh frozen plasma, PLTconc: platelet concentrate, ACC: aorta cross clamp * *p* < 0.05.

**Table 3:** Postoperative morbidity and mortality across three NLR tertiles.

	First Tertile (*n* = 111)	Second Tertile (*n* = 111)	Third Tertile (*n* = 112)	*p*-Value
HOD (days)	14.02 ± 13.97	20.29 ± 21.72	19.65 ± 15.31	0.005 *
ICU (days)	6.04 ± 61.20	3.49 ± 5.64	6.43 ± 9.25	0.805
Reintubation	7 (6.4%)	11 (10.3%)	22 (20.0%)	0.007 *
MV > 24 h	7 (6.3%)	12 (10.8%)	34 (30.4%)	<0.001 *
ICU readmission	7 (6.3%)	13 (11.7%)	9 (8.0%)	0.344
Reopen for bleeding	1 (0.9%)	2 (1.8%)	8 (7.1%)	0.046 *
CVA	2 (1.8%)	1 (0.9%)	2 (1.8%)	1.000
New RRT	2 (1.8%)	6 (5.4%)	4 (3.6%)	0.358
Pulmonary Cx	8 (7.2%)	22 (19.8%)	27 (24.1%)	0.002 *
Infection	4 (3.6%)	8 (7.2%)	17 (15.2%)	0.007 *
Wound infection	0	1 (0.9%)	2 (1.8%)	0.776
AKI	50 (45.0%)	45 (40.9%)	55 (50.0%)	0.399
MI	1 (0.9%)	3 (2.7%)	3 (2.7%)	0.707
In-hospital mortality	3 (2.7%)	10 (9.0%)	15 (13.4%)	0.015 *
1-month mortality	2 (1.8%)	7 (6.3%)	12 (10.7%)	0.023 *
1-year mortality	3 (2.7%)	10 (9.0%)	16 (14.3%)	0.009 *

Values are presented as means ± standard deviations or number of patients (%). HOD: hospital day, ICU: intensive care unit, MV: mechanical ventilation, CVA: cerebrovascular accident, RRT: renal replacement therapy, Pulmonary Cx: pulmonary complication, AKI: acute kidney injury, MI: myocardial infarction * *p* < 0.05.

**Table 4:** Logistic regression analysis for the predictors of 1-year mortality after AAA open repair.

	Univariate OR (CI)	*p*-Value	Multivariate OR (CI)	*p*-Value
**All Patients**				
Age	1.027 (0.979–1.076)	0.277		
Rupture	2.859 (1.131–7.225)	0.026	2.706 (1.097–6.673)	0.031 *
Smoking	1.255 (0.524–3.005)	0.610		
HTN	0.619 (0.258–1.483)	0.282		
DM	1.096 (0.369–3.258)	0.869		
CAOD	1.483 (0.590–3.724)	0.402		
CVA	1.356 (0.436–4.214)	0.599		
CKD	0.430 (0.050–3.663)	0.440		
NLR	1.077 (1.007–1.152)	0.030	1.085 (1.016–1.159)	0.015 *
PLR	0.996 (0.990–1.002)	0.181	0.995 (0.989–1.001)	0.103
MPV	0.710 (0.496–1.016)	0.061	0.716 (0.504–1.019)	0.063
**Non-rupture**				
Age	1.010 (0.941–1.085)	0.782		
Smoking	0.886 (0.229–3.428)	0.860		
HTN	0.408 (0.113–1.465)	0.169	0.403 (0.125–1.295)	0.127
DM	0.623 (0.071–5.440)	0.668		
CAOD	1.252 (0.310–5.052)	0.752		
CVA	1.875 (0.360–9.756)	0.455		
CKD	1.863 (0.201–17.258)	0.584		
NLR	1.040 (0.954–1.133)	0.376		
PLR	1.003 (0.996–1.009)	0.440		
MPV	0.773 (0.440–1.357)	0.730		
**Rupture**				
Age	1.025 (0.955–1.101)	0.495		
Smoking	0.852 (0.528–6.494)	0.336		
HTN	0.888 (0.244–3.225)	0.857		
DM	1.568 (0.368–6.676)	0.543		
CAOD	1.791 (0.474–6.767)	0.390		
CVA	0.890 (0.162–4.887)	0.893		
CKD	0.000 (0.000)	0.999		
NLR	1.139 (1.020–1.271)	0.020	1.144 (1.031–1.271)	0.012 *
PLR	0.988 (0.977–1.000)	0.047	0.986 (0.975–0.998)	0.017 *
MPV	0.617 (0.367–1.037)	0.069	0.616 (0.374–1.013)	0.056

Values are presented as odds ratios (95% confidence intervals). HTN: hypertension, DM: diabetes mellitus, CAOD: coronary artery occlusive disease, CVA: cerebrovascular accident, CKD: chronic kidney disease, NLR: neutrophil to lymphocyte ratio, PLR: platelet to lymphocyte ratio, MPV: mean platelet volume * *p* < 0.05.

**Table 5:** Laboratory data according to the presence of rupture.

	Non-Rupture (*n* = 236)	Rupture (*n* = 98)	*p*-Value
Preop WBC (/μL)	8249.36 ± 2931.44	14,442.14 ± 5960.32	<0.001 *
Preop neutrophil (/μL)	5569.29 ± 2947.05	12,154.97 ± 5628.89	<0.001 *
Preop lymphocyte (/μL)	1824.74 ± 822.26	1374.01 ± 670.37	<0.001 *
Preop NLR	4.10 ± 4.75	11.17 ± 7.90	<0.001 *
Preop Hb (g/dL)	12.85 ± 2.13	9.70 ± 2.47	<0.001 *
Preop Hct (%)	38.47 ± 6.13	29.47 ± 7.30	<0.001 *
Preop PLT (/μL)	217,017.52 ± 71,052.99	168,704.08 ± 78,781.86	<0.001 *
Preop MPV (fL)	9.26 ± 1.19	9.59 ± 1.24	0.023 *
Preop PLR	142.22 ± 84.14	155.52 ± 111.62	0.291
Preop PT (INR)	1.06 ± 0.16	1.35 ± 0.42	<0.001 *
Preop procalcitonin (ng/mL)	0.18 ± 0.84	0.32 ± 0.39	0.245
Preop CRP (mg/dL)	19.34 ± 35.91	30.08 ± 57.75	0.130
Preop BNP (pg/mL)	201.50 ± 355.12	130.09 ± 189.65	0.149
Preop lactate (mmol/L)	0.97 ± 0.41	4.45 ± 2.66	<0.001 *

Values are presented as means ± standard deviations (%). NLR: neutrophil to lymphocyte ratio, Hb: hemoglobin, Hct: hematocrit, PLT: platelet, MPV: mean platelet volume, PLR: platelet to lymphocyte ratio, PT: prothrombin time, CRP: C-reactive protein, BNP: B type natriuretic peptide * *p* < 0.05.

## Data Availability

Due to internal institutional restrictions, raw data would remain confidential and would not be shared but would be made available on reasonable request and with the permission of the institution where the data were generated.
